# Advances in engineered bacteria vaccines for enhancing anti-cancer immunity

**DOI:** 10.20517/mrr.2024.75

**Published:** 2025-01-15

**Authors:** Wu Liu, Wenjing Chen, Yujie Cai, Yanhua Tang, Tingtao Chen

**Affiliations:** ^1^The Second Affiliated Hospital, Jiangxi Medical College, Nanchang University, Nanchang 330008, Jiangxi, China.; ^2^School of Pharmacy, Jiangxi Medical College, Nanchang University, Nanchang 330008, Jiangxi, China.; ^3^Department of Cardiovascular Surgery, The Second Affiliated Hospital, Jiangxi Medical College, Nanchang University, Nanchang 330008, Jiangxi, China.; ^4^Jiangxi Province Key Laboratory of Bioengineering Drugs, The MOE Basic Research and Innovation Center for the Targeted Therapeutics of Solid Tumors, School of Pharmacy, Jiangxi Medical College, Nanchang University, Nanchang 330031, Jiangxi, China.; ^5^National Engineering Research Center for Bioengineering Drugs and the Technologies, Institute of Translational Medicine, Jiangxi Medical College, Nanchang University, Nanchang 330031, Jiangxi, China.; ^#^Authors contributed equally.

**Keywords:** Engineered bacteria, antitumor, neoantigenic, immunotherapy

## Abstract

Advances in synthetic biology have enabled the development of tumor-targeted live bacterial therapeutics. In a recent study published in *Nature*, Redenti *et al.* engineered *Escherichia coli* Nissle 1917(EcNc^Δlon/ΔompT/LLO+^ nAg), which exploits the advantages of living medicines to deliver arrays of tumor-specific neoantigenic epitope in optimal environments, thereby providing a novel strategy for developing effective and durable cancer immunotherapies.

Engineered bacteria have been explored for tumor-targeted therapy for over a century. In recent years, advances in synthetic biology tools have enabled these bacteria to release therapeutic agents directly at the tumor site, enhancing the effectiveness of antitumor therapy. Engineered bacteria retain the probiotic properties of the original strain and can also serve as vectors to express various antitumor molecules, including cytokines, cytotoxic agents, immunomodulators, tumor-associated antigens, antibodies, pro-drug enzymes, siRNAs, and other therapeutic compounds^[1]^. Additionally, these bacteria can be programmed to control their growth spatially and temporally, ensuring the release of therapeutic agents only upon sensing specific target molecules, thereby improving the efficacy of bacterial cancer therapy^[2]^. Gujrati *et al.* constructed biomimetic nanoparticles (OMV^Mel^) for tumor therapy with OMV^[3]^. The engineered bacteria can secrete OMV containing a large amount of melanin with high photothermal conversion efficiency, mediating photoacoustic (PA) imaging and photothermal treatment of 4T1 breast cancer. In addition, the OMV^Mel^-induced PA signal lasts for at least 24 h, enabling relatively long-term tumor monitoring. Mimee *et al.* developed a microcapsule equipped with a microelectronic device and genetically engineered bacteria to rapidly detect gastrointestinal disorders such as gastric hemorrhage, ulcers, inflammatory bowel disease, and colon cancer in a minimally invasive manner^[4]^. The integration of multiple transcripts into engineered bacteria can compound detection and analysis to improve the robustness of diagnostic tests. Overall, engineered bacteria are becoming increasingly important in tumor benign and malignant diagnosis, therapeutic effect prediction, and clinical biomarker identification.

In this study, the authors engineered the probiotic *Escherichia coli* Nissle 1917 (EcN) as a tumor vaccine platform and optimized the production and cytoplasmic delivery of neoantigenic peptide arrays to induce specific, effective, and durable systemic antitumor immunity^[5]^. *In vivo* experiments demonstrated that both intratumoral and intravenous administration of the engineered neoantigen vaccine, as well as prophylactic injection, significantly inhibited CT26 tumor growth. Importantly, EcNc^Δlon/ΔompT/LLO+^ nAg^19^ was detected within the tumor microenvironment, while bacteria present in the circulatory system were progressively cleared. Simultaneously, robust activation of tumor-specific CD4^+^/CD8^+^ T cells dominated the antitumor immune response, accompanied by a marked reduction in both CD4^+^ regulatory T cells and suppressive tumor-associated macrophages (TAMs) within the tumor. Moreover, the researchers observed significantly elevated levels of interleukin-12p70 within the tumor, indicating that the T_H_1 immune response was effectively activated. The same therapeutic effects were reproduced in a more aggressive tumor model (B16F10 melanoma), where the engineered bacteria containing neo-tumor antigens also played a role in preventing cancer recurrence. Thus, the engineered bacterial strains developed by the authors exhibited enhanced immunogenicity and reduced tumor immunosuppression. Furthermore, personalized therapeutic strategies allow the vaccine to more precisely target the patient’s tumor, minimizing damage to normal tissues.

This approach parallels the work of the Stanford team^[6]^; however, the genetic engineering modifications of EcN have been executed in a more refined manner, resulting in several advantages: (1) Strain Construction: As a prokaryote, the production of antigens is not as quantitative and efficient as in eukaryotic cells. Therefore, the authors optimized the structure of the synthetic neoantigen constructs by pioneering the removal of cryptic plasmids and the deletion of the Lon and OmpT proteases. This led to an up to 80-fold increase in neoantigen synthesis compared to the parental EcN strain, establishing a new paradigm for synthesizing antigens in engineered bacteria and suggesting that antigenic targets can be expressed in large quantities to enhance therapeutic efficacy; (2) Immunoconjugation: As an emerging therapeutic approach, the authors developed a microbial drug delivery system by integrating the prototypical gene of the neoantigen construct (NeoAgp), which enhances susceptibility to phagocytosis and promotes greater neoantigen uptake by antigen-presenting cells (APCs), thereby facilitating MHC-II class antigen presentation. This allows for improved and more efficient activation of CD4^+^ and CD8^+^ T cells. Simultaneously, the expression of listeriolysin O (LLO) enables better cytoplasmic access for the presentation of recombinantly encoded neoantigens via MHC-I class molecules, thereby promoting a T helper cell 1 (T_H_1)-type immune response; (3) Clearance: As a live bacterial agent, there is a risk of infection if it cannot be effectively cleared during clinical use. In this study, the authors reduced the viability and biofilm formation of EcNc^Δlon/ΔompT/LLO+^ nAg in the bloodstream, achieving a 1,000-fold increase in sensitivity to blood clearance. This improvement enhances safety during systemic administration, allowing the bacteria to be rapidly cleared after fulfilling their therapeutic role.

However, there are two sides to every issue. (1) Given that clinical treatment involves extended medication regimens, repeated high-dose administrations of the same strain may activate immune clearance mechanisms, thereby limiting the bacteria’s ability to colonize the tumor long enough to achieve therapeutic concentrations. This challenge is also encountered by lysogenic bacteria and viruses. The engineered EcNc developed by the authors has only one set of systems, potentially leading to immune tolerance. To address this, developing a variety of engineered probiotic species could enhance efficacy and reduce the risk of tolerance. Several genera, including *Salmonella*, *Clostridium*, *Bifidobacterium*, *Proteus*, and *Lactobacillus*, have demonstrated immunostimulatory effects, suggesting their potential as tumor-targeting vectors^[1]^; (2) Moreover, sequencing tumors and employing bioinformatics to identify their unique neoantigens demand advanced technological support. A key challenge is that tumor cells may lose target antigens over time, which could weaken the therapeutic effect of the engineered bacteria, leading to drug resistance and disease recurrence; (3) The system developed by the authors removes the cryptic plasmid and enhances the ability to produce antigens; however, there is potential for the transmission of resistance genes. Cryptic plasmids, characterized by their high copy number and genetic stability, have a broad influence on human gut microbes and can affect host metabolic pathways, drug resistance, and other biological properties; (4) Despite the authors’ demonstration of high blood clearance, the complexity and variability of clinical oncology patients, coupled with the greater variability of combination therapy with chemotherapeutic agents, mean that the potential risk of infection during clinical translation should not be overlooked; (5) Meanwhile, if the safety and efficacy of the vaccine are clinically validated, and individual differences and cost issues are addressed, it will be more suitable for clinical application.

Overall, this report describes the construction of the engineered probiotic *Escherichia coli* Nissle 1917(EcNc^Δlon/ΔompT/LLO+^ nAg), which takes advantage of living medicines to deliver arrays of tumor-specific neoantigenic epitope in optimal environments. This approach provides a new strategy for the development of effective and durable cancer immunotherapies. Looking ahead, probiotic-based tumor neoantigen delivery vectors hold great promise as efficient and widely applicable cancer therapies, potentially marking a significant breakthrough in precision oncology.

## DECLARATIONS

### Copyright

© The Author(s) 2025.
